# Refusing to Treat Sexual Dysfunction in Sex Offenders

**DOI:** 10.1017/S0963180116000712

**Published:** 2017-01

**Authors:** THOMAS DOUGLAS

**Keywords:** conscientious objection, conscientious refusal, treatment refusal, sex offenders, testosterone therapy, sexual dysfunction, agent-relativity, complicity, discrimination, statistical discrimination, indirect discrimination

## Abstract

This article examines one kind of conscientious refusal: the refusal of healthcare professionals to treat sexual dysfunction in individuals with a history of sexual offending. According to what I call the *orthodoxy*, such refusal is invariably impermissible, whereas at least one other kind of conscientious refusal—refusal to offer abortion services—is not. I seek to put pressure on the orthodoxy by (1) motivating the view that either both kinds of conscientious refusal are permissible or neither is, and (2) critiquing two attempts to buttress it.

In February 2013, I was approached by a group of urologists from the Boston Medical Center regarding a policy of treatment refusal that they were considering for adoption. One of the chief conditions that these urologists were involved in treating was male sexual dysfunction—a condition that frequently consists of impotence, reduced sexual desire, inability to orgasm, or a combination of these symptoms. The urologists had become aware that some patients presenting for treatment for this condition were individuals with a history of sex offending. They were concerned about this because it seemed to them that treating sexual dysfunction in these individuals could increase the likelihood that they would reoffend, especially because treatment often involved administration of testosterone, a hormone that typically increases sex drive.

It seemed to the urologists that there should be a system whereby forensic psychiatrists specialising in sex offenses would assess past sex offenders and offer guidance on whether treatment for sexual dysfunction could safely be provided. However, no such system existed and they believed it unlikely that one would be introduced in the short-to-medium-term future. They were, therefore, considering taking matters into their own hands by introducing a policy of (1) investigating all new patients for a history of sexual offending, via direct questioning, review of medical records, and/or screening against sex offender registries; and (2) declining to treat sexual dysfunction in any individuals found to have such a history.

## Two Arguments for Treatment Refusal

The urologists have subsequently introduced this policy.^1^ They offered two main arguments in support of it, which I present here in slightly reconstructed form.

The first argument was an appeal to consistency. The urologists noted that chemical castration—the hormonal suppression of testosterone—was widely used in the United States and Europe to facilitate the rehabilitation of sex offenders, often without the offender’s consent. It seemed to them that, insofar as their own policy of treatment refusal consisted in withholding testosterone treatment, what they were doing was functionally equivalent to such chemical castration: it involved restricting testosterone levels and, therefore, libido, with the goal of preventing sexual recidivism. To this it might be added that there is at least one respect in which refusing testosterone treatment to sex offenders seems less problematic than chemical castration, at least in its nonconsensual forms: it involves withholding treatment rather than enforcing it. It is standardly thought within medical ethics that physicians enjoy a wider prerogative to refuse treatments that they do not support than to enforce treatments that they do support.

The second argument was more straightforward: the urologists believed that treating sexual dysfunction could reasonably be expected to bring about future sex offenses and that this gave them a sufficient moral reason to decline to offer such treatment.

In this article I set aside the first argument, both because it does not clearly involve any appeal to the permissibility of conscientious refusal, the focus of this special issue, and because it is not, in my view, persuasive (there are many disanalogies between the use of chemical castration within criminal justice or forensic psychiatry and the withholding of testosterone treatment in clinical urology). Instead, I will focus on the second argument, which seems to me more promising and which is, I think, best construed as an argument for conscientious refusal.

## Empirical Doubts

Although I believe that the urologists’ second argument is more promising than their first, it does also face some significant difficulties—difficulties that the urologists acknowledged: there is limited evidence to support the empirical speculation that treating sexual dysfunction in sex offenders can reasonably be expected to contribute to future sex offenses.^2^

This is not to say that the speculation is groundless. There is *some* reason to believe that certain forms of treatment might diminish recidivism in certain groups of sex offenders. Testosterone treatment is arguably the intervention for which this rationale is strongest; testosterone is an important driver of libido, which is thought to be an important driver of at least some kinds of sexual offending.^3^ Moreover, there is evidence—albeit of limited quality—that pharmacologically suppressing testosterone can substantially diminish sexual recidivism in sex offenders deemed clinically suitable for such treatment,^4^ suggesting that administration of testosterone might have the opposite effect. Treatments for impotence could also theoretically increase the risk of recidivism, insofar potential offenders are deterred from offending by a fear of impotence.^5^

There is, therefore, something to be said for the urologists’ hypothesis. But the considerations just mentioned fall well short of providing a robust empirical basis for the putative link between treating sexual dysfunction and recidivism. Moreover, for many offenders, offending is motivated by nonsexual factors, and there is little reason to suppose that treating sexual dysfunction would increase risk of recidivism in these offenders. Similarly, for many offenders (for example, those whose only offense occurred a long time previously), there is, in any case, a low risk of reoffending.^6^ These factors suggest that any substantial antirecidivist effect of treatment refusal will be limited to a subgroup of offenders.

In addition to the effects of treatment on the *likelihood* of recidivism, we might be concerned about its effects of the severity of recidivism or the degree of harm that it inflicts. Unfortunately, the situation here is similarly unclear. On the one hand, there is some evidence that penetrative sexual attacks tend to cause greater psychological and physical harm than nonpenetrative attacks,^7^ suggesting that treatments for impotence might have a harm-promoting effect. However, and militating in the opposite direction, there is also some evidence that sexual offenders may become more violent if they experience impotence during an attack.^8^

## Assumptions and Qualifications

To evade these empirical issues, I will henceforth focus only on testosterone therapy—not other forms of treatment for sexual dysfunction—and will assume that testosterone therapy does increase recidivism risk at least for some identifiable classes of offenders and types of sexual dysfunction. (I take “recidivism risk” here to be a metric of both likelihood and likely severity of reoffending.)

I will also make a number of further assumptions, which I capture in the form of a hypothetic policy—a policy that the urologists might have adopted, although it is slightly different from the one that they in fact adopted. In this hypothetic policy, which I will call *testosterone refusal*, urology staff check all new patients against publicly available sex offender registries and collect as much forensic history from patients as they are willing to provide. They then input this information into the best available actuarial risk assessment tool^9^ and refuse testosterone therapy for all patients for whom such therapy can reasonably be estimated to result in *one or more additional serious sex offenses*.

One might think that whether this policy is morally justified will depend on the legal status of the refusal; it is plausible that there are (defeasible) moral reasons to comply with the law, at least in a more-or-less just legal system. To evade this issue, I will assume that the testosterone refusal is neither legally required nor legally prohibited.

One might also think that the moral justifiability of the policy will depend on the motives for which it is pursued. To evade this issue as well, I will assume that testosterone refusal is motivated by a concern to protect potential future victims of sex offenses, a motive which I take to be beyond moral reproach.

Finally, I will introduce a limitation on the scope of my argument. I will not consider whether testosterone refusal is, all things considered, morally justified. Rather, I will simply compare its moral features—including the strength of the case for its moral justifiability—to the paradigmatic policy of conscientious refusal in medicine, which I call *abortion refusal*. I take abortion refusal to be the policy, adopted by a healthcare professional, of refusing to perform (certain kinds of) abortion. I assume that this refusal is motivated by the belief that performing the relevant kind of abortion would be morally wrong in virtue of it involving the killing of a fetus. And I assume that abortion refusal is neither legally required nor legally prohibited.

## The Orthodoxy

What view would most healthcare professionals and healthcare ethicists take regarding abortion refusal? As I will henceforth put it, what would be the *dominant view* on abortion refusal? I believe it would be that abortion refusal is morally permissible, at least if done in certain ways (for example, if coupled with referral to another physician who is willing to perform an abortion). The view that abortion refusal is permissible if coupled with referral is often termed the *conventional compromise*.^10^ By contrast, I think the dominant view regarding *testosterone* refusal would be that it is *not* permissible, even under those same conditions. This suggestion is supported by the fact that there has been virtually no discussion of testosterone refusal or similar policies in the ethical literature on conscientious refusal and by the fact that, unlike abortion, testosterone therapy (whether for sex offenders or others) is not among the interventions explicitly picked out by the statutory “conscience clauses” that explicitly authorize conscientious refusal of specified medical interventions.^11^ (Both facts would be somewhat surprising if testosterone refusal were taken to be permissible by most healthcare professionals and ethicists.) It is also supported by the fact that policies such as testosterone refusal are not generally excluded from a widely held more general norm against the use of forensic history to inform decisions regarding the provision or withholding of treatment. Commenting on a case in which a criminal offender sought a heart transplant, bioethicist Arthur Caplan maintained that “[f]or me, it’s open and shut. . . . It is absolutely wrong to make judgments about past behavior, criminal conduct, moral worth, indictments, charges or convictions.”^12^ Caplan is here arguably expressing a widely held view regarding the place of forensic history in medical decisionmaking, and this view is not normally qualified so as to exclude policies such as testosterone refusal.^13^

I therefore believe the dominant views regarding abortion refusal and testosterone refusal to be that (1) the former is sometimes permissible, but (2) the latter is not. I henceforth refer to the conjunction of these two views as, for want of a better term, *the orthodoxy*. In what follows, I examine whether and, if so, how the orthodoxy can be given a satisfying rationale.

## Grounds for Doubting the Orthodoxy

Before exploring possible rationales, however, I wish to briefly motivate my doubts about the orthodoxy. One reason to doubt its correctness is that there are plausible ways of accounting for the moral permissibility of abortion refusal that seem also to entail the permissibility of testosterone refusal. For example, it seems to me plausible that, if and when abortion refusal is permissible, it is permissible because it satisfies two conditions.

*Condition 1*: The doctor (I use “doctor” to refer to any healthcare professional) reasonably believes that refusal to perform the intervention in question would, at least given the like refusal of others, avert a grave moral wrong.

(The thought here is that doctors reasonably believe that, by refusing to perform the intervention, they are doing their bit to prevent the occurrence of a grave moral wrong.)

*Condition 2*: The magnitude of the harm imposed on the patient by the doctor’s refusal to perform the intervention falls below some acceptable threshold.

Arguably, these conditions are, or are in the same ballpark as conditions that are, individually necessary and jointly sufficient conditions for the permissibility of conscientious refusal in medicine. Moreover, defenders of abortion refusal can argue with some plausibility that both of these conditions hold in relation to abortion refusal, or at least, certain variants of that policy. Some doctors who refuse to perform abortions *do* believe that they are thereby averting (or would avert, given the like refusal of others) a grave moral wrong, and given uncertainty regarding the moral status of the embryo and fetus, this belief can, arguably, be reasonable. Moreover, if abortions are refused under certain conditions (for example, those in which patients can easily access other healthcare professionals who *are* willing to perform abortions) and in certain ways (for example, with referral to such professionals), their refusal may be relatively costless for the patient seeking the abortion, meaning that the second condition is also likely to be satisfied.

However, it is, I think, equally plausible that these conditions could be satisfied in relation to testosterone refusal. Condition 1 clearly holds in relation to that policy. One of the assumptions built into testosterone refusal is that it is reasonable to believe that universal refusal to provide the testosterone treatment would prevent a sex offense, and it is clearly reasonable to believe that the commission of a sex offense constitutes a grave moral wrong. In fact, it seems not merely reasonable to believe this, but rationally required—it seems unreasonable to believe otherwise.

It is less clear that Condition 2 is satisfied in relation to testosterone refusal; however, it is arguably just as plausible that it is satisfied in relation to testosterone refusal as that it is satisfied in relation to abortion refusal. To see this, it is important to note that there are two main kinds of harm that can be imposed on a patient by a doctor’s refusal to provide some treatment: (1) the costs of accessing the treatment elsewhere, and (2) the costs of going without the treatment if it cannot be accessed elsewhere. It should be expected that both of these costs will normally be lower in relation to testosterone refusal than in relation to abortion refusal. Given that testosterone refusal is rare, and standard practice when treating sexual dysfunction is not to enquire into sexual forensic history, it should in general be relatively easy for sex offenders refused treatment to find treatment elsewhere. In addition, it seems reasonable to assume that the costs of going without treatment for sexual dysfunction will typically be lower than those of bringing an unwanted pregnancy to term and then either raising an unwanted child or adopting the child out.

## Defending the Orthodoxy

I have been outlining some grounds for doubting that testosterone refusal and abortion refusal differ in their moral permissibility. I now want to consider how someone might respond to these doubts. In what follows, I consider in detail two attempts to drive a moral wedge between these two cases and thus rescue the orthodoxy.

Each of these attempts involves revising Conditions 1 and 2—the conditions that I posited for permissible conscientious refusal in medicine—such that they can accommodate the permissibility of abortion refusal without also implying the permissibility of testosterone refusal. In each case, I will assess the attempt by considering whether the revised conditions (1) can indeed accommodate the permissibility of abortion refusal, or at least those variants of it that are widely thought permissible, (2) imply the impermissibility of testosterone refusal, and (3) are independently plausible, for example, because they are consistent with a satisfying moral theory or are able to accommodate commonly held intuitions about other cases.

Before turning to these two attempts, however, I shall briefly explain why I set aside what may appear to be two more obvious ways of defending the orthodoxy.

One way of defending the orthodoxy would be to argue that the killing of a fetus constitutes a graver wrong than the perpetration of a serious sex offense. There are certain moral views according to which the killing of a fetus is, or is morally equivalent to, the murder of an innocent person, and the murder of an innocent person is arguably more gravely wrong than the perpetration of a sex offense. Therefore, there will perhaps be some (very high) threshold level of gravity that we could specify in Condition 1 such that abortion refusal could reasonably be taken to pass the threshold, whereas testosterone refusal could not.

This, however, seems to me to be an unpromising line of argument, for two reasons. First, some sex offenses also involve the murder of an innocent person; therefore, the defender of testosterone refusal could respond to it by arguing that testosterone refusal is permissible under the same conditions as abortion refusal, where those conditions involve a requirement that the wrong that the refuser seeks to avert is (or is equivalent to) the murder of an innocent person.

Second, *if* abortion refusal is ever morally permissible, it is very plausible that it is permissible even in cases in which the refuser does not believe, or does not reasonably believe, that the killing of a fetus is morally equivalent to the murder of an innocent person. Consider the case of a doctor who reasonably believes that, although abortion is less grave than murder, it does involve the killing of a being of significant moral status. Suppose this doctor believes that performing an abortion is comparable in the gravity of the wrong to the infliction of one week of moderate pain on an innocent person. Most supporters of abortion refusal would, I think, find it permissible for *this* doctor to refuse to perform abortions. However, it is implausible that infliction of one week of moderate pain on an innocent person is a graver wrong than the perpetration of a serious sex offense against a person. Therefore, if abortion refusal is accepted in such cases, one will be hard pressed to exclude testosterone refusal on the grounds that the wrong at stake in testosterone refusal is insufficiently grave.

A second initial attempt to buttress the orthodoxy would appeal to a difference in the *certainty* of the putative wrongdoing averted by treatment refusal. Given the like refusal of others, abortion refusal will *certainly* prevent a grave moral wrong (if everyone refuses to perform an abortion, no fetus-killing occurs). By contrast, given the like refusal of others, testosterone refusal *can be reasonably be expected to* prevent a grave moral wrong (a future sex offense), but it does not do so with certainty. The relationship between testosterone therapy and sex offending is merely statistical and it is always possible that an offender will reoffend (or not reoffend) regardless whether he is given testosterone treatment.

It might be thought that this makes a moral difference to the permissibility of conscientious refusal. It might be argued, for example, that Condition 1 should be replaced by:

*Condition 1’*: The doctor reasonably believes that his or her (or universal) refusal to perform the intervention would *certainly* avert a grave moral wrong.

Again, however, this strikes me as an unpromising attempt to buttress the orthodoxy.

First, in the absence of any further story about why certainty is morally significant, the revision to Condition 1 suggested seems *ad hoc.* Why, if an instance of treatment can reasonably be *expected* to avert a grave moral wrong, should it make a difference whether it will do so with certainty? The only potentially satisfying answer that I can see to this question appeals to the idea that the lack of certainty in testosterone refusal means that, in refusing testosterone treatment, one engages in a form of statistical discrimination, and I will deal with that possibility separately later.

Second, contrary to the story that I outlined, it is not clear to me that Condition 1’ *can* accommodate the permissibility of abortion refusal. Though there is no empirical uncertainty in the abortion refusal case—it is certain that the abortion will not occur if everyone refuses to perform it—there arguably *is* moral uncertainty—it is plausibly uncertain whether abortion is in fact gravely wrong, since there are credible moral views according to which it is not. Therefore, it is uncertain whether even universal refusal to perform an abortion would avert a grave moral wrong.

To avoid this second difficulty, Condition 1’ would need to be further modified so as to distinguish between empirical and moral uncertainty. But this, I think, would merely exacerbate the first problem—the problem of “ad hocness*.*” Setting aside the issue of statistical discrimination, it seems to me that it would be puzzling if that empirical uncertainty undermined the case for treatment refusal, although moral uncertainty did not.

Let me turn, then, to consider two attempts to buttress the orthodoxy that are, I think, more promising.

## Attempt One: Agent Relativity

One difference between abortion refusal and testosterone refusal is that, in abortion refusal, the putative wrong that doctors seeks to avert is a wrong that they would otherwise commit themselves (viz. the killing of a being of significant moral status). By contrast, in testosterone refusal, the wrong (viz. a future sex offense) that doctors seek to avert is a wrong committed by someone else—the patient.

For those who accept that morality can be agent relative, this difference may be an important one. It might be thought that doctors have more reason to avoid their own wrongdoing than to avert the wrongdoing of others, so that the case for conscientious refusal is stronger where the wrong averted is one committed by the doctor. Perhaps it could even be claimed that conscientious refusal is *only* justified in such cases. To capture this view, we could replace Condition 1 above with, for example:

Condition 1*: The doctor reasonably believes that refusing to perform the intervention in question will prevent *him or her from committing* a grave moral wrong.

It might seem that this condition will hold regarding many variants of abortion refusal, but that it fails to hold in relation to testosterone refusal, because the sex offenses that testosterone refusal is intended to prevent will not be committed by the doctor.

Importantly, however, even though doctors will not themselves commit a sex offense, if they provide testosterone therapy, they may commit another wrong: namely, the wrong of foreseeably elevating the risk that the patient commits a sex offense. Therefore, there remains scope to argue that testosterone refusal could satisfy Condition 1*. It would do so in cases in which doctors implementing testosterone refusal reasonably believe that, were they to provide testosterone therapy, they would at least elevate the risk that their patient commits a sex offense and that this risk-elevation would amount to a grave moral wrong.

It might be thought that Condition 1* will be satisfied only in a vanishingly small range of cases, however. For example, it might be argued that elevating the risk that one’s patient commits a sex offense will only very rarely, if ever, qualify as a *grave* moral wrong. At least two factors might be invoked to discount its graveness.

First, it might be thought that there is a morally significant difference between committing a wrong that consists in bringing it about (or elevating the risk) that someone else commits a wrong and committing a *primary* wrong: a wrong that is not in this or any other way mediated by the wrongdoing of another agent. Arguably, it is less wrong, other things being equal, to bring it about that (or elevate the risk that) someone else commits a wrong than to commit a primary wrong. This distinction might be defended by appealing to a more general moral distinction between being an *accomplice* to wrongdoing and committing a primary wrong. Bringing about (or elevating the risk of) a wrong is one way—although perhaps not the only way—of becoming an accomplice to a wrong committed by another.

Second, it might be argued that the gravity of the putative wrong committed by the doctor who prescribes testosterone is diminished by the fact that any harm that the doctor thereby brings about to the victim of a future sex offense is clearly unintended, whereas the harm that a doctor causes to the fetus in the course of performing an abortion is intended. Some believe that it is, other things being equal, less seriously wrong to unintentionally but foreseeably bring about harm than to intentionally bring about harm.^14^

It should be noted, however, that each of these mitigating factors is controversial, and there are widely held and somewhat credible moral theories—for example, most forms of consequentialism—that would reject them or hold that the mitigation they offer is quite minor. It therefore seems that it could be reasonable for a doctor to believe that these mitigating factors have little or no force, and that expectably bringing about a sex offense therefore constitutes a grave moral wrong. The doctor might, after all, reasonably accept consequentialism! This suggests that testosterone refusal could satisfy Condition 1*, provided that the doctor refusing to provide testosterone therapy does indeed hold the relevant reasonable beliefs.

In reply, it might be argued that healthcare workers are subject to role-specific moral requirements (and permissions)—to what I will call *medical* morality. And it is arguably clear that this medical morality *does* assign substantial weight to at least the first of the two distinctions that I mentioned above—the distinction between complicity and primary wrongdoing.

For example, the supposedly supreme principle of medical ethics—“above all do no harm”—is sometimes interpreted in such a way that it applies only or at least more strongly to harms inflicted directly *by the doctor*. Therefore, a doctor who knowingly prescribes an unsafe sleeping pill might be thought to violate the principle, whereas a doctor who prescribes a sleeping pill knowing that the patient is likely to (unsafely) share it with his wife might be thought not to violate it. On at least some interpretations, then, the principle appears to implicitly incorporate a distinction between complicity and primary wrongdoing.

This distinction also plays a role in the thinking of many healthcare professionals and ethicists about conscientious refusal. Many accept the conventional compromise, according to which conscientious refusal to perform an abortion is justified, but conscientious refusal to *refer* for an abortion is not.^15^ The difference between performance and referral that is normally invoked to justify this view is that referral involves “only” a wrong of complicity whereas performance involves primary wrongdoing.^16^

Medical morality, as commonly understood, therefore does appear to discount wrongs of complicity relative to primary wrongdoing, at least in some cases. It might seem clear, then, that according to medical morality, any wrong committed by a doctor who provides testosterone therapy to a sex offender is mitigated by the fact that the doctor will not be the primary perpetrator of that wrong. Perhaps it is mitigated to such a degree that it could not be invoked to justify conscientious refusal.

There is, however, scope to doubt whether medical morality discounts wrongs of complicity to the degree required for this argument to succeed.

First, one might question whether widely held views about medical morality, such as the conventional compromise regarding conscientious refusal to perform abortion, accurately reflect the *true* role-specific moral considerations bearing on doctors. It has been argued, in my view convincingly, that conventional medical morality gives *too little* weight to wrongs of complicity—that true medical morality would give such wrongs greater weight.^17^

Second, it might be argued that even conventional medical morality is somewhat equivocal on the significance of the complicity–primary wrongdoing distinction. Although the principle of nonmaleficence and the conventional compromise regarding conscientious refusal to perform abortions do seem premised on the idea that wrongs of complicity should be substantially discounted relative to primary wrongs in some contexts, in other contexts, conventional medical morality appears to reject any such discounting, or at least to accept that wrongs of complicity may be grave. For example, a number of international medical ethics codes prohibit the involvement of medical professionals in torture even if their involvement would be as accomplices—for example, through helping to develop safer or more effective torture techniques—not principal agents.^18^ Conventional medical morality seems to accept that wrongs of complicity can be sufficiently grave to justify conscientious refusal in some cases. Consider the recent case in which a number of Australian doctors refused to discharge patients back to what they regarded as inhumane refugee camps.^19^ The wrongdoing that the doctors believed they would have committed had they discharged the patients was a wrong of complicity; the principal wrong was one perpetrated by the Australian State. Nevertheless, I would speculate that conventional medical morality would be at least qualifiedly supportive of conscientious refusal in this case.

It seems, then, that there is, at the very least, significant room for doubt regarding whether an appeal to the complicity–primary wrongdoing distinction can establish that the harm averted in the testosterone refusal case is insufficiently grave to justify conscientious refusal.

## Attempt Two: Discrimination

A second attempt to justify the impermissibility of testosterone refusal without conceding the impermissibility of abortion refusal would appeal to an antidiscrimination requirement. It might be argued that testosterone refusal is impermissible because it involves a kind of wrongful discrimination. (I take wrongful discrimination to be discrimination that infringes the rights of the person discriminated against, although I leave open the possibility that such infringements may sometimes be justified, for example, because the rights infringed against are not absolute.) It might seem that testosterone refusal involves wrongful discrimination because it singles out a particular group of patients—namely, sex offenders, or sex offenders with a certain risk of reoffending—for less favourable treatment than others.

By contrast, abortion refusal arguably does not involve wrongful discrimination, because it does not involve singling out any group of patients for unfavorable treatment. Although it is true that the burdens of abortion refusal fall only on women, it might be argued that women are not formally *treated less favorably* than men by doctors who follow this policy. Presumably doctors who refuse to perform abortions for women would also refuse to perform abortions for men, should these be a possibility; therefore, it might seem that they are formally treating women and men equivalently, refusing all requested abortions. (I will revisit this point later.)

What I am suggesting here is that we may need to add, to our conditions for permissible conscientious refusal in medicine, something like the following:

*Condition 3*: Refusal to perform the intervention in question does not wrongfully discriminate against the person to whom the intervention is refused.

This condition would arguably rule out testosterone refusal but not abortion refusal.

Should Condition 3 be accepted, however? And can this condition really distinguish between abortion refusal and testosterone refusal? I will focus on the second of these questions. It might seem that, contrary to what I have just suggested, Condition 3 cannot distinguish these two policies because, in fact, *neither* abortion refusal nor testosterone refusal involves unjustified discrimination. On the dominant analyses of wrongful discrimination, an instance of unfavorable treatment constitutes wrongful discrimination (and perhaps even discrimination *simpliciter*) only if it is based on the victim’s being a member of a certain kind of group. For example, some would argue that unfavourable treatment is (wrongfully) discriminatory only when the group is socially salient in the sense that “perceived membership of it is important to the structure of social interactions across a wide range of social contexts,” as in the case of ethnic and gender groups,^20^ or where group membership is unchosen.^21^ Unfavorable treatment of sex offenders on the basis of their being sex offenders arguably meets neither of these conditions.

Still, it might be maintained that testosterone refusal *is* wrongfully discriminatory, and if some influential accounts of wrongful discrimination cannot accommodate this, so much the worse for those accounts. One way of motivating this claim would appeal to a similar case in which a charge of wrongful discrimination seems difficult to resist. Suppose an eye surgeon introduces a policy of screening all new patients for a history of sex offending and refuses to treat new-onset blindness in all patients with such a history in whom this refusal can be expected to prevent at least one serious sex offense. Call this policy *surgery refusal.* Surgery refusal seems clearly wrong, and plausibly on the basis that it is discriminatory. Moreover, it might seem that if surgery refusal involves wrongful discrimination, then, given the similarities between the policies, testosterone refusal must too. At least, consideration of surgery refusal might seem to create some pressure to accept the view that testosterone refusal also involves wrongful discrimination.

How can we make sense of the view that testosterone refusal involves wrongful discrimination, however? As I noted, it does not seem to satisfy the conditions for wrongful discrimination offered by the dominant analyses. I can see two somewhat plausible possibilities.

First, it might be argued that testosterone refusal involves a wrongful kind of *statistical* discrimination. Statistical discrimination involves the members of one group being treated less favorably than others on the basis of statistical evidence that members of that group differ from those others on some dimension.^22^ For example, racial profiling in airport security is often thought to involve statistical discrimination because it involves treating members of certain racial groups less favorably than others (by singling them out for additional security checks) on the basis of statistical evidence that members of those groups are more likely than others to carry out terrorist attacks or otherwise threaten security. Likewise, testosterone refusal arguably involves treating certain sex offenders less favorably than others (by depriving them of testosterone therapy) on the basis of statistical evidence that they are more likely than other people to commit sex offenses in the future.

Although the wrongfulness of statistical discrimination is controversial,^23^ it is sometimes thought wrongful, even in (some) cases in which it does not rely on socially salient or unchosen group membership, for example because it involves failing to treat people as individuals,^24^ imposes costs on some innocent individuals in virtue of the choices or behavior of other individuals who happen to be members of the same group,^25^ or fails to give individuals the chance to disprove the statistical generalization and, therefore fails to respect their autonomy.^26^

Second, it might plausibly be maintained that healthcare professionals fall under some role-specific antidiscrimination requirement that is wider in scope than the antidiscrimination requirement that applies more generally. That is, it may be that a wider range of forms of unfavorable treatment qualify as wrongfully discriminatory when the putative discriminator is, and is acting as, a healthcare professional. In particular, it might be thought that the antidiscrimination requirement that applies to healthcare professionals is not restricted to unfavourable treatment based on socially salient or unchosen group membership. For example, I think many would judge that healthcare professionals fall under a requirement that they not treat some patients less favorably than others on the basis of any group membership that is *medically irrelevant*. This sort of view would help to explain why it would generally be thought wrong for health professionals to, for example, refuse to treat patients with moral views that they find distasteful.

It is not entirely clear how the distinction between medically relevant and medically irrelevant group memberships is to be drawn, but intuitively, group memberships that are strongly predictive of medical need or prognostic of poor treatment response are medically relevant, whereas group memberships that are of no or little diagnostic or prognostic relevance are not. (Of course, whether a particular group membership is medically relevant may depend on the context in or purpose for which it is used. For example, an individual’s being an abuser of alcohol may be medically relevant in the context of the allocation of livers for transplantation, but not in the context of funding counseling for depression.) Intuitively, it might seem that the group “sex offenders” is not medically relevant with respect to decisions to provide testosterone therapy and that testosterone refusal (and for that matter surgery refusal) therefore violates this broad-scope antidiscrimination requirement to which healthcare professionals may be subject.

Although I have no firm view about the wrongfulness of statistical discrimination or about the scope of a medicine-specific antidiscrimination requirement, it seems to me that appealing to one or both of these ideas will be the most promising way of resisting the permissibility of testosterone refusal while preserving the permissibility of abortion refusal, and I am open to the possibility that this appeal may succeed. However, I do want to end by raising some worries about it.

My primary worry is that, contrary to what the proponent of this appeal must maintain, it is not clear to me that *abortion* refusal can plausibly be thought to avoid the charge of wrongful discrimination.

As noted, the costs of abortion refusal *do* disproportionately fall on members of a certain group—namely, women (and even more disproportionately on certain subgroups of women, for example, young, poor, and geographically isolated women). Although it is arguable that these groups are not formally treated unfavourably (abortions are refused to all), it is not obvious that this rules out the presence of wrongful discrimination. Many authors recognize a category of acts that are *indirectly* discriminatory. The basic idea is that indirect discrimination occurs when treatment is not (directly) discriminatory in form but is relevantly like discrimination in its outcome.^27^ Typically a policy qualifies as indirectly discriminatory because it formally treats individuals unfavourably on the basis of some group membership that is not itself a basis for a charge of wrongful direct discrimination, but is correlated with one that is. For example, suppose that the group “individuals with a low level of education” is not socially salient in the way required for unfavourable treatment of low-educated individuals to count as wrongful direct discrimination, whereas the group “Hispanic Americans” is socially salient in the required way. Therefore, it would normally be wrongfully directly discriminatory to turn someone down for a job on the basis of that person’s being a Hispanic American, but it would not be wrongfully directly discriminatory to turn that person down for a job on the basis of the person being poorly educated. However, suppose that there is a strong correlation between being Hispanic American and being poorly educated. Then it might be held that it is wrongfully, although *in*directly, discriminatory (or potentially so, depending on further features of the case) to exclude persons of low education from a certain type of job on the basis that they are poorly educated.

Similarly, abortion refusal may qualify as indirectly discriminatory because abortion is refused on the basis that the patient is a member of a group (for example, the group of people seeking abortions) that is highly correlated with a group (e.g., young, poor women) against whom unfavourable treatment would be directly and wrongfully discriminatory.

Now, the moral status of indirect discrimination is disputed,^28^ but probably the dominant view is that it is less morally problematic than direct discrimination. Therefore, perhaps it remains possible to separate abortion refusal and testosterone refusal by holding that the antidiscrimination requirement on healthcare workers only applies, or applies with greater force, to direct discrimination, and that direct discrimination occurs only with testosterone refusal, not with abortion refusal. In fact, it might be thought that allowing the prohibition of discrimination within medicine to extend to indirect discrimination will have absurd implications, because in almost every case that medical treatment is declined, for example out of concerns for efficient use of resources, the treatment refusal decision will be based on a characteristic that is correlated with some group membership that could serve as a basis for wrongful direct discrimination.

It is worth noting, however, that indirect discrimination *is* sometimes regarded as objectionable in medicine.

For example, some object to the use of rational procedures based on Quality or Disability Adjusted Life-Years (QALYs/DALYs) on the grounds that they differentially disadvantage old people, who generally have shorter life expectancies and therefore less to gain from a particular treatment.^29^ Such objections are arguably best understood as maintaining that QALY-based rationing constitutes indirect discrimination against older people. There may be scope to argue that a medical antidiscrimination requirement applies to some forms of indirect discrimination, perhaps including that involved in abortion refusal.

Perhaps more importantly, it also seems possible that abortion refusal could be *directly* discriminatory. To see this, suppose that an obstetrician refuses abortions not to all women who seek them, but only to those whose pregnancies were not the result of contraceptive failure or rape. The doctor believes that abortion is wrong only when the woman is responsible for her pregnancy, and that the necessary responsibility does not obtain when the pregnancy was the result of rape or contraceptive failure. In this case, the obstetrician explicitly and formally treats women pregnant for reasons other than rape and contraceptive failure less favorably than women pregnant as a result of rape or contraceptive failure; however, it is doubtful that this distinction qualifies as medically relevant*.* It therefore seems that the obstetrician will fall afoul of our imagined broad-scope antidiscrimination requirement. Nevertheless, if any variants of abortion refusal are permissible, it seems plausible that this variant, perhaps suitably further qualified, could be among them. This suggests that either healthcare professionals *are not* subject to a broad antidiscrimination requirement that rules out unfavorable treatment on nonmedical grounds, or that, if they are subject to such a requirement, it is not sufficiently strong to render impermissible all forms of conscientious refusal that violate the requirement. This in turn casts doubt on whether *testosterone* refusal is impermissible by virtue of violating such a requirement.

## Concluding Thoughts

In this article, I have sought to challenge the orthodoxy—that is, the view that abortion refusal is under certain conditions permissible, whereas testosterone refusal (even under those same conditions) is not. I first outlined a prima facie promising set of necessary and sufficient conditions for permissible treatment refusal in medicine, noting that these conditions plausibly apply equally to abortion refusal and testosterone refusal. I then turned to consider two attempts to buttress the orthodoxy.

The first attempt appealed to the differing relationship between healthcare professionals and the putative wrong that they seek to avert through treatment refusal under the two policies. In abortion refusal, the primary putative wrong—the killing of the fetus—is one that would be committed by healthcare professionals themselves. By contrast, in testosterone refusal, the primary wrong—a future sex offense—is one that would be committed by someone else—namely, the patient.

I argued, however, that testosterone refusal might prevent the healthcare professional from committing a different wrong: the wrong of (expectably) bringing about the occurrence of a serious sex offense. Moreover, I challenged the view that medical morality substantially discounts wrongs of complicity relative to primary wrongs. I thereby sought to support the view that it could be reasonable to believe that the wrong of (expectably) bringing about a serious sex offense is a grave one, and thus one that might ground permissible treatment refusal.

The second attempt appealed to the thought that testosterone refusal involves wrongful discrimination whereas abortion refusal does not. I allowed that testosterone refusal might indeed involve such discrimination by virtue of the fact that it involves treating certain sex offenders less favorably than other patients on the basis of a medically irrelevant difference between them, or by virtue of its involving wrongful statistical discrimination. However, I then suggested that abortion refusal—including forms of abortion refusal that would widely be thought permissible—may also involve wrongful discrimination, either because it involves indirectly discriminating against women (or the particular subgroups of women on whom the burdens of abortion refusal chiefly fall), or because it involves direct discrimination through selective refusal of abortions to certain subgroups of women.

What do my arguments, if successful, imply? If I have considered all plausible bases for the orthodoxy, they imply that the orthodoxy has no rational basis. Of course, it might be argued that the widespread acceptance and intuitive plausibility of the orthodoxy itself provides a sufficient basis for accepting it, even in the absence of a rational grounding. I am not, however, inclined to this view myself. I therefore suggest that we ought to reconsider the orthodoxy, by reconsidering the dominant views about abortion refusal, testosterone refusal, or both.

## Notes

1. Some of the reasoning behind the introduction of the policy is described in PhillipsEA, RajenderA, DouglasT, BrandonAF, MunarrizR. Sex offenders seeking treatment for sexual dysfunction—ethics, medicine, and the law. The Journal of Sexual Medicine 2015;12(7):1591–1600.2606094210.1111/jsm.12920PMC6962056

2. See note 1, Phillips et al. 2015 at 1594–5, 1597.

3. There is also some evidence that sildenafil (Viagra) and other nontestosterone treatments for sexual dysfunction can increase libido. See, for discussion, note 1, Phillips et al. 2015, at 1595.

4. MaletzkyBM, TolanA, McFarlandB. The Oregon Depo-Provera program: A five-year follow-up. Sexual Abuse: A Journal of Research and Treatment 2006;18(3):303–16.1698889310.1177/107906320601800308

5. See note 1, Phillips et al. 2015, at 1595.

6. See note 1, Phillips et al. 2015, at 1596.

7. Pinhas-HamielO, Modan-MosesD, Herman-RazM, ReichmanB. Obesity in girls and penetrative sexual abuse in childhood. Acta Paediatrica 2009;98:144–7;1879883210.1111/j.1651-2227.2008.01044.x Canton-CortesD, CortesMR, CantonJ. Child sexual abuse, attachment style, and depression: The role of the characteristics of abuse. Journal of Interpersonal Violence 2015;30:420–36.2495813310.1177/0886260514535101

8. JonesJS, RossmanL, WynnBN, OstovarH. Assailants’ sexual dysfunction during rape: Prevalence and relationship to genital trauma in female patients. The Journal of Emergency Medicine 2010;38:529–35.1923287210.1016/j.jemermed.2008.09.037

9. Actuarial tools for the prediction of sexual recidivism do already exist, with the STATIC-99/R being the most widely used of these. See HansonRK, HarrisAJR, HelmusL, ThorntonD. High-risk sex offenders may not be high risk forever. Journal of Interpersonal Violence 2014;29:2792–813.2466425010.1177/0886260514526062

10. BrockD. Conscientious refusal by physicians and pharmacists: Who is obligated to do what, and why?. Theoretical Medicine and Bioethics 2008;29:187–200;1875637510.1007/s11017-008-9076-y WicclairM. Conscientious Objection in Health Care: An Ethical Analysis. Cambridge: Cambridge University Press; 2011. See also: CantorJ, BaumK. The limits of conscientious objection—may pharmacists refuse to fill prescriptions for emergency contraception? New England Journal of Medicine 2004;351:2008–12.1552572810.1056/NEJMsb042263

11. Pope TM. Legal briefing: Conscience clauses and conscientious refusal. *The Journal of Clinical Ethics* 2009;21(2):163–76.

12. Kolata G. Inmate fears death because prison won’t finance transplant. *The New York Times* February 5, 1994; available at http://www.nytimes.com/1994/02/05/us/inmate-fears-death-because-prison-won-t-finance-transplant.html (last accessed 10 May 2016).

13. Moreover, even those who reject Caplan’s view and argue for the exclusion of criminal offenders from certain forms of medical treatment have often held that this exclusion should be implemented by the state, not individual (groups of) healthcare professionals. They have not seen such exclusion as a matter for individual conscientious refusal. See, for example, SchneidermanLJ, JeckerNS. Should a criminal receive a heart transplant? Medical justice vs. societal justice. Theoretical Medicine 1996;17(1):33–44899264510.1007/BF00489739, at 34. I do suspect that many healthcare professionals and ethicists might wish to allow for the refusal of testosterone treatment in cases in which, through no effort on his or her part, a urologist becomes aware that a patient has a history of sexual offending. However, I suspect that, even among those who endorse this view, the dominant view would be that the urologist should not actively enquire into such a history, but should treat without seeking out this forensic knowledge.

14. See, for the classic statement of this view, Aquinas T. Summa Theologica II-II, Q. 64, art. 7, “Of killing.” In: Baumgarth WP, Regan RJ, eds. *On Law, Morality, and Politics*. Indianapolis/Cambridge: Hackett Publishing Co; 1988:226–7.

15. See note 10, Brock 2008; Wicclair 2011; Cantor, Baum 2004.

16. See, for example, note 10, Brock 2008.

17. See, for example, BaylesMD. A problem of clean hands, refusal to provide professional services. Social Theory and Practice 1979;5(2):165–81; Minerva F. Conscientious objection, complicity in wrongdoing, and a not-so-moderate approach. *Cambridge Quarterly of Health Care Ethics* 2017;26(1); Devolder K. Complicity after the fact: Japan’s wartime medical atrocities. Presentation given at *Analytic Bioethics in Europe Conference*, Ghent University, Ghent, Belgium, May 28–29, 2014.

18. See, for example, World Medical Association Declaration of Tokyo – Guidelines for Physicians Concerning Torture and other Cruel, Inhuman or Degrading Treatment or Punishment in Relation to Detention and Imprisonment. World Medical Association 1975, revised in 2005 and 2006; available at http://www.wma.net/en/30publications/10policies/c18/ (last accessed 22 Feb 2016), which specifies that physicians must not “participate in the practice of torture or other forms of cruel, inhuman or degrading procedures,” “provide any premises, instruments, substances or knowledge to facilitate the practice of torture,” or “be present during any procedure during which torture or any other forms of cruel, inhuman or degrading treatment is used or threatened,” See also Position Statement on Identification of Abuse and Misuse of Psychiatry. World Psychiatric Association 1998, Principle 5 (“Psychiatrists shall not . . . use medical knowledge for interrogation, persuasion or torture”); available at http://www.wpanet.org/uploads/Latest_News/News_from_WPA_Sections/attach%2003.pdf (last accessed 22 Feb 2016); and Torture, death penalty and participation by nurses in executions: ICN Position. International Council of Nurses 1998, last revised 2012; available at http://www.icn.ch/images/stories/documents/publications/position_statements/E13_Torture_Death_Penalty_Executions.pdf (last accessed 22 Feb 2016): “nurses should play no voluntary role in any deliberate infliction of physical or mental suffering and should not participate, either directly or indirectly, in the preparation for and the implementation of executions.”

19. Hatch P, Ireland J, Booker C. Royal Children’s Hospital doctors refuse to return children to detention. *The Age (Melbourne)* October 11, 2005; available at http://www.theage.com.au/victoria/royal-childrens-hospital-doctors-refuse-to-return-children-to-detention-20151010-gk63xm.html (last accessed 25 Feb 2016).

20. See, for this view, Lippert-RasmussenK. The badness of discrimination. Ethical Theory and Moral Practice 2006;9:167–85; Lippert-RasmussenK. Born Free and Equal? A Philosophical Inquiry into the Nature of Discrimination. New York: Oxford University Press; 2013. The quoted passage is from Lippert-RasmussenK. Nothing personal: On statistical discrimination. Journal of Political Philosophy 2007;15(4):385–403, at 386.

21. KahlenbergRD. The Remedy: Class, Race, and Affirmative Action. New York: Basic Books; 1997.

22. See, for a recent definition, note 20, Lippert-Rasmussen, 2007, at 386–9.

23. See especially note 20, Lippert-Rasmussen 2007.

24. See, for example, Wasserman DT. The morality of statistical proof and the risk of mistaken liability. *Cardozo Law Review* 1991;13:935–76, esp. at 943; Miller D. *Principles of Social Justice*. Cambridge, MA: Harvard University Press; 1999:168–9.

25. See, for example, Colyvan M, Regan HM, Ferson S. Is it a crime to belong to a reference class? *Journal of Political Philosophy* 2001;9(2):168–81., at 175–6. For critical discussion of this view, see note 20, Lippert-Rasmussen 2007, at 397–9.

26. See note 24, Wasserman 1991, at 942–3. Walter Sinnott-Armstrong (personal communication, 24 Nov 2015) also suggested to me that the wrongness of testosterone refusal might lie in its failure to give offenders the chance to redeem themselves, though he did not put the point in terms of statistical discrimination.

27. For a recent definition, see Lippert-Rasmussen K. Indirect discrimination is not necessarily unjust. *Journal of Practical Ethics* 2014;2(2):33–57, at 37–38: “A policy, practice or act is indirectly discriminatory against a certain group if, and only if: 1) it neither explicitly targets nor is intended to disadvantage members of the group (the no-intention condition); 2) it disadvantages members of the group (the disadvantage condition); and 3) the relevant disadvantages are disproportionate (the disproportionality condition).”

28. For an argument that indirect discrimination is not necessarily wrongful, see note 27, Lippert-Rasmussen 2014.

29. See, for example, Harris J. QALYfying the value of life. *Journal of Medical Ethics* 1987;13(3):117–23, esp. at 119; Harris J. It’s not NICE to discriminate. *Journal of Medical Ethics* 2005;31(7):373–75.

